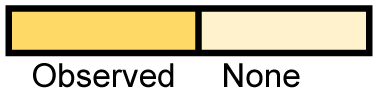# Agonist antibodies for cancer immunotherapy: History, Hopes and Challenges

**DOI:** 10.1158/1078-0432.CCR-23-1014

**Published:** 2024-05-01

**Authors:** Sean H. Lim, Stephen A. Beers, Aymen Al-Shamkhani, Mark S. Cragg

**Affiliations:** 1 Antibody and Vaccine Group, Centre for Cancer Immunology, School of Cancer Sciences, University of Southampton Faculty of Medicine, Southampton, SO16 6YD, UK; 2 Institute for Life Sciences, University of Southampton, Southampton, SO17 1BJ, UK

## Abstract

Immunotherapy is amongst the most promising new treatment modalities to arise over the last two decades; antibody drugs are delivering immunotherapy to millions of patients with many different types of cancer. Initial success with antibody therapeutics came in the form of direct targeting or cytotoxic antibodies, such as rituximab and trastuzumab, which bind directly to tumor cells to elicit their destruction. These were followed by immunomodulatory antibodies, that elicit anti-tumor responses by either stimulating immune cells or relieving tumor-mediated suppression. By far the most successful approach in the clinic to date has been relieving immune suppression, with immune checkpoint blockade now a standard approach in the treatment of many cancer types. Despite equivalent and sometimes even more impressive effects in pre-clinical models, agonist antibodies designed to stimulate the immune system have lagged behind in their clinical translation. In this review we document the main receptors that have been targeted by agonist antibodies, consider the various approaches that have been evaluated to date, detail what we have learnt and consider how their anti-cancer potential can be unlocked.

## Introduction

It is well recognised that the immune system is central to our health and well-being and that when its ability to maintain homeostasis is disturbed, pathology typically follows. Cancer is considered by many to be a direct consequence of immune-suppression and that for cancer to develop it must avoid elimination by the immune system, causing Hanahan and Weinberg to include it amongst their revised Hallmarks of Cancer.(1) Therefore, it is unsurprising that boosting anti-tumor immune responses and/or overcoming tumor immune-suppression are key goals of antibody immunotherapy for cancer. In order to achieve these goals and thereby benefit patients, detailed understanding of immune regulation is required. Immune cells have evolved to protect the host against pathogens and malignancy whilst avoiding destruction of self-tissues and commensals. To achieve this balance, immune cells like T cells only become fully functional after receiving a primary signal through their antigen receptor (T cell receptor; TCR) and a secondary antigen-independent signal known as costimulation.(2) As such, T cell costimulation is tightly regulated by both intrinsic factors including the expression of inhibitory receptors (CTLA-4 and PD-1) and through extrinsic mechanisms involving Toll-like receptor and regulatory T cell modulation of costimulatory ligands on antigen presenting cells.(3–5)

Costimulatory receptors belong to either the immunoglobulin superfamily (IgSF) or the TNF receptor superfamily (TNFRSF), with both contributing to the regulation of immunity. Considering T cells as an example, although the signal transduction mechanisms of these two costimulatory receptor families differ, both types of receptors transmit signals that combine with signalling pathways downstream of the TCR/CD3 complex to instigate quantitative and qualitative changes that culminate in increased T cell proliferation and survival, metabolic fitness, as well as differentiation into effector cells.(6–8) A similar set of co-stimulatory interactions serves to modulate the fate and function of many other immune cells including B cells and dendritic cells.

Given the essential role that costimulatory receptors play in the generation of successful immune responses their targeting has been proposed as an approach to stimulate anti-tumor responses.(9–11) Although non-antibody agonists, including fusion proteins, cyclic peptides and aptamers have been tested with variable success, the majority of agents destined for clinical development are antibodies or antibody-like molecules. Developing agonist antibodies that mimic the effects of natural membrane-bound costimulatory ligands, however, has proved considerably more challenging than developing checkpoint inhibitors directed against CTLA-4 and PD-1. This is in part due to differences in the valency and geometry of receptor binding between the natural ligand and antibody. For example, membrane anchoring of trimeric TNFRSF ligands such as CD27L, CD40L, 4-1BBL and OX40L is obligatory for optimal receptor signalling, likely due to the formation of higher order structures that facilitate assembly of the signalosome.(12) In contrast, trimeric GITRL, TL1A and lymphotoxin αβ are fully functional in solution.(12) Recent structural studies of the GITRL:GITR complex suggest that receptor homodimerization could enhance avidity for GITRL and also lead to formation of higher order structures upon receptor binding to soluble trimeric GITRL.(13) Thus, for TNFRSF ligands that function in solution this mechanism could provide an alternative way to assemble optimal receptor:ligand complexes on the cell surface.

Despite these challenges, agonist antibodies targeting the IgSF receptors CD28(14), ICOS(15) and CD96(16) as well as those targeting several members of the TNFRSF have been generated.(17) Interestingly, the agonistic activity of many of these antibodies requires Fc gamma receptor (FcγR) mediated hyper-crosslinking emphasising the importance of higher order receptor clustering for optimal signal transduction(17); see later sections.

Prediction of the therapeutic activity of immune agonists in human subjects is often complex and requires careful consideration of multiple factors including knowledge of immune cell subset specific expression as well as detailed analysis of receptor biology. In this regard, studies of inborn errors of immunity which are available for a subset of costimulatory receptors/ligands, including CD27/CD70(18), CD28(19), ICOS(20), 4-1BB/4-1BBL(21–23) and OX40(24) are highly informative and should be considered together with findings from studies in animal models. Meanwhile, detailed mechanistic studies in animal models are providing insights into the effects of combining agonists with checkpoint blockers to better understand how these treatments synergise for improved immunotherapy.(25–27) Despite equivalent and sometimes more profound effects in pre-clinical models(9,11) agonist antibodies have lagged behind in their clinical translation compared to checkpoint blockers. Below, we review the agents (focussing mainly on unmodified antibody isotypes and canonical antibody formats directed to CD40, 4-1BB, OX40, GITR, CD27 and DR5) that have been assessed clinically to date, detail areas where progress is being made and provide reasons for optimism for the future of this antibody class.

## Mechanisms underpinning agonist activity

**Figure 1:**
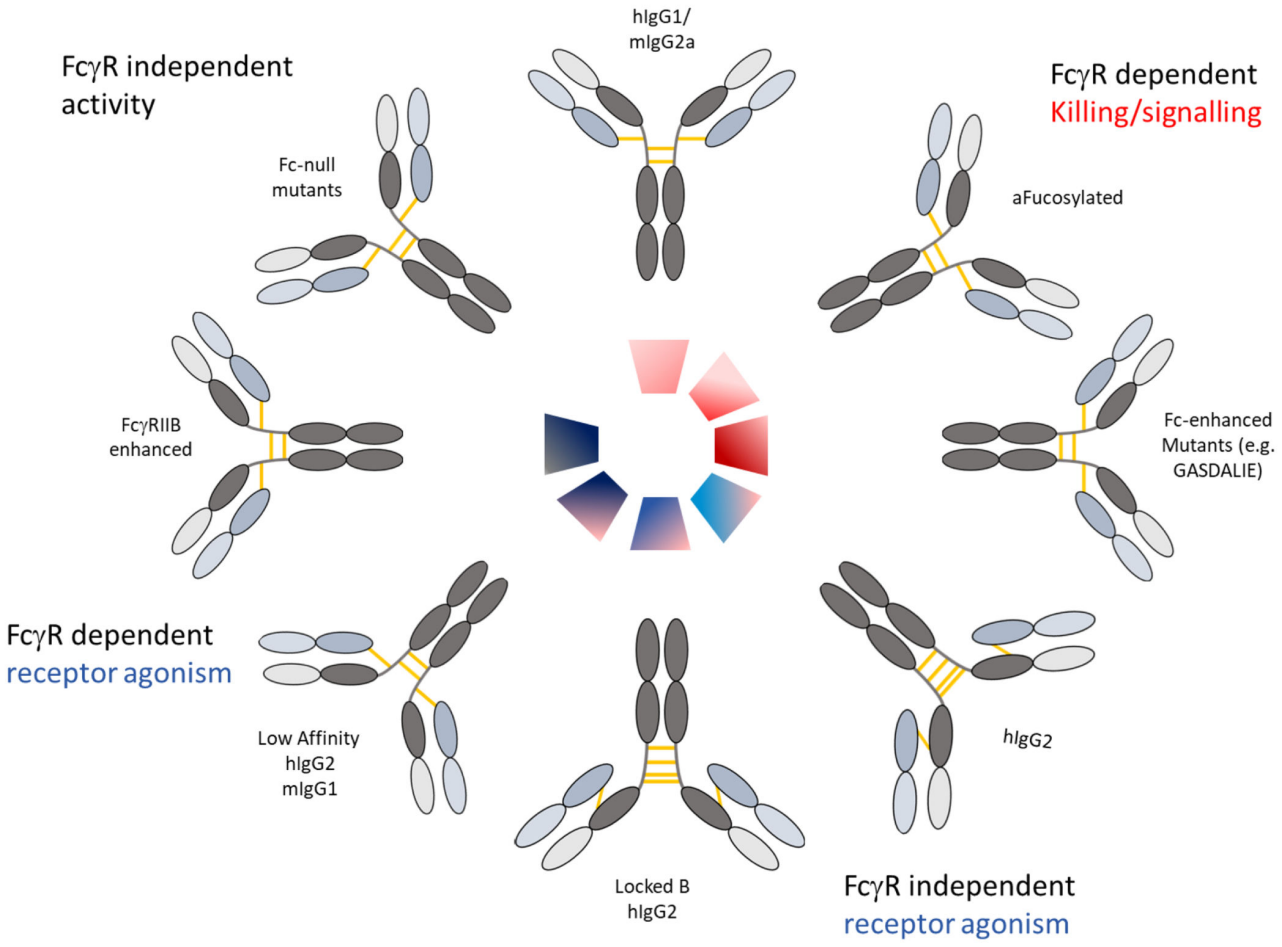
Current formats of monospecific IgG used to elicit direct targeting and/or receptor agonism. Target cell killing (red) and/or receptor agonism (blue) can be evoked using various isotype and formats of mAb; clockwise from top centre illustrating wild-type hIgG1 and mIgG2a as powerful native isotypes capable of delivering direct cell killing. Afucosylation can augment this activity through enhanced affinity to FcγRIII or mFcγRIV, respectively as can amino-acid modifications in the Fc(61), where additional FcγRs can be engaged more effectively. Native hIgG2 can evoke FcγR-independent agonism, via IgG2B forms, which can also be generated through C-S hinge mutations resulting in “locked” IgG2B forms. Reduced affinity mAb in mIgG1 and hIgG2 isotypes can also elicit higher agonism, which can be similarly achieved through interaction with FcγRIIB, again through modifications to the Fc. Finally, Fc-null mutants can be generated through Fc mutations and/or deglycosylation which can allow mAb to deliver their activities (e.g. receptor blocking or agonism) independently of FcγR interaction.

Whereas the “rules” for isotype selection for direct targeting or cytotoxic mAb are clear with hIgG1/3 in humans and mIgG2a/c in mice selected as having the highest activating:inhibitory FcγR engagement ratio and cytotoxic activity(28,29), they are not immediately evident for agonist antibodies. As described above, most agonist antibodies differ from their direct targeting/cytotoxic counterparts, by being optimally effective with isotypes that preferentially engage the inhibitory FcγR(30–34); or avoid FcγR binding entirely as summarised in Figure 1.

**Figure 2:**
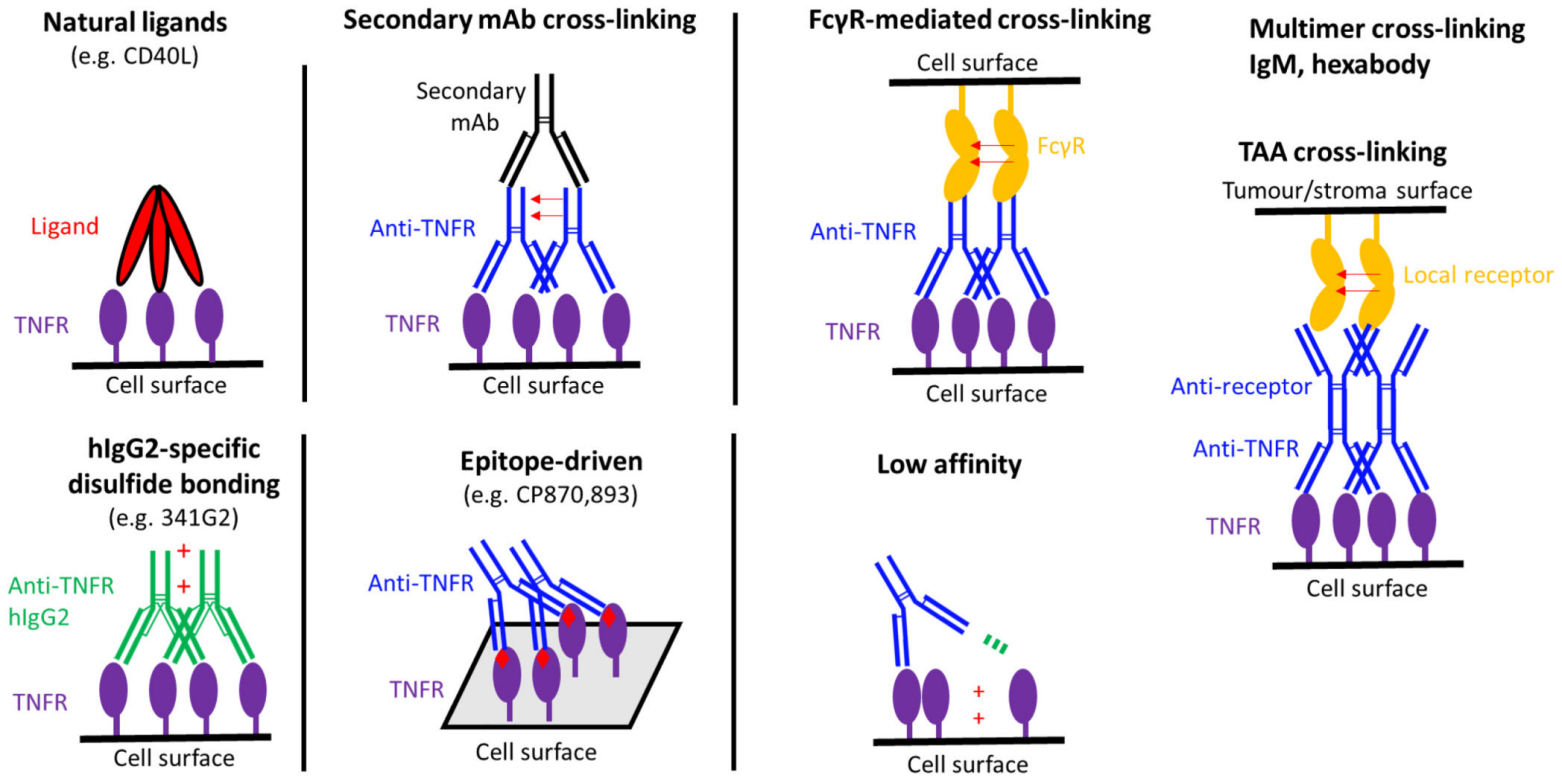
Means to evoke agonism. Multiple means through which receptor agonism can be evoked; clockwise from top left illustrating natural ligands, secondary “hyper” cross-linking often used in vitro, FcγR-mediated cross-linking, the related cross-linking achieved with another (non-FcγR) receptor such as a tumour associated antigen (TAA), low affinity mediated agonism, epitope-driven and hIgG2(B) mediated. Not shown are the multiple different multivalent methods through which cross-linking mediated agonism might be delivered including through tetra- and hexa-valent mAb and molecules, as well as IgM hexabody

In the murine system, the optimal isotype is mIgG1, which, following binding by mFcγRII enhances receptor cross-linking, mimicking the action of the natural ligand.(35) In all cases that have been reported, agonistic activity correlates with cell surface receptor clustering(36), with non-agonists or antagonists inert in this respect. Other means of providing such cross-linking provide scope for increasing the activity of agonist antibodies. As summarised in Figure 2, and described in part below, these strategies involve experimental methods such as secondary antibody cross-linking or antibody immobilisation/coating(37) in addition to means to increase receptor multimerization, exemplified by tetramerization(38) or hexamerisation(39,40) using antibodies, antibody-like molecules or ligand;antibody hybrids.

For the canonical bivalent antibodies, based upon the experiences of targeting TNFRSF members in the mouse system, the most straightforward path to translation would have been to select a mIgG1-like human isotype. However, given the mouse:human differences in FcγR expression patterns and isotype binding profiles, there is no direct human equivalent to mIgG1.(41) Empirically, hIgG2 has been shown to elicit the greatest level of activity for several human agonist antibodies.(42) This was first demonstrated for anti-hCD40 agonists leveraging powerful B cell stimulation and antigen-specific CD8 T cell expansion, followed by similar evidence with mAb directed to 4-1BB, CD28(35), OX40(36) and CD27(43). The hIgG2 isotype was even able to convert anti-CD40 antagonists into maximally potent agonists.(44) Perhaps the most surprising property of the hIgG2 isotype was its ability to deliver agonism largely independently of FcγR.(35,44,45)

The agonistic capacity of hIgG2 relates to its unique hinge as evidenced by hinge-swaps with other isotypes.(42,44) The hIgG2 hinge contains additional cysteines which can undergo disulfide-shuffling to adopt different configurations, with IgG2A and IgG2B representing the extremes.(46,47) The IgG2B form is most agonistic (42,44), correlating with a more compact and less flexible mAb conformation; this is also true across IgG isotypes with hIgG2 the most compact and agonistically active and the highly flexible hIgG3 least agonistic.(48) A range of orthogonal biophysical techniques showed that agonism was linked to the presence of a disulphide cross-over between opposing heavy and light chains in engineered IgG2B forms(49), which imparted the more compact, less flexible agonist confirmation. The current model is that this more compact and less conformationally diverse molecular arrangement restricts receptor mobility in the plasma membrane, promoting more efficient receptor clustering and therein activation.

However, selection of hIgG2 alone is not sufficient to impart agonism for all antibodies and it is clear that epitope is also an important determinant. For example, although mAb binding to membrane distal domain CRD1 were shown to be agonistic for anti-CD40 mAb, and mAb directed to other CRDs less active, there was still a spectrum of activity for CRD1-targeting mAb. This was true for both mIgG1 and hIgG2 isotypes, with some mAb largely inert in any isotype, indicating that both epitope and isotype combine to mediate the net level of agonism for a given mAb.(45) These same studies also revealed that rare antibodies such as CP870,893 are agonistic independent of isotype.(35,44,45) Similar observations have been made for anti-4-1BB mAb where urelumab was demonstrated to be active in multiple isotypes (albeit more agonistic as hIgG2), whereas utomilumab was robustly inactive in all isotypes assessed.(45,50). This reveals that certain antibodies target rare epitopes that facilitate agonism without reliance on Fc or FcγR-dependent properties, although it has been shown that they may in certain contexts be augmented by FcγR engagement.(50–52)

A further determinant of agonist mAb activity is affinity. Following initial observations in a series of anti-CD95 mAb that demonstrated poorer apoptosis induction following affinity maturation(53), the impact of affinity on immunomodulatory antibodies was recently investigated.(54) Using antibody series targeting either CD40 or 4-1BB it was shown that reduced affinity increased receptor agonism, until a point where binding to the receptor was no longer detectable. Agonist activity still required bivalency, did not need FcγR interaction (but could be enhanced by it), was evoked in multiple isotypes (mIgG1 and hIgG2) and was correlated with increased receptor clustering on the cell surface. The hypothesis proposed is that lower affinity mAb bind bivalently, enabling them to bring 2 receptors together, before one F(ab) releases its receptor (due to high off-rate) and binds a third molecule and pulls it into the receptor complex before the released receptor has been able to migrate away; thereby nucleating larger receptor arrays that can better deliver agonistic signals. These findings add to our understanding of the various ways in which agonism can be achieved, as summarised in Figure 2.

## Impact of the tumor micro-environment

There is a further complication to consider in delivering efficacy with agonist antibodies - the tumor microenvironment (TME). The TME comprises a wide array of host factors such as blood vessels, lymphatics, extracellular matrix, fibroblasts, innate and adaptive immune cells, and soluble factors in addition to tumor cells. Considering the context of this review, co-stimulatory receptors are largely expressed on myeloid (CD40) or T cell populations (OX40, 4-1BB, GITR and CD27). Consequently, these targets will be impacted differently dependent upon the varying TME and may require different mechanisms of action to induce therapeutic responses.

For example, CD40 expression is largely restricted to myeloid populations within the TME. Agonist mAb have been shown to act either directly through macrophages inducing T cell independent anti-tumor protection(55) or indirectly via dendritic cell cross-presentation leading to T cell mediated anti-tumor clearance.(56) Myeloid cells, particularly macrophages which can make up a significant proportion of the TME alter their phenotype and activation status in response to environmental cues. In the context of the TME there have been numerous studies observing that the inhibitory FcγRIIB becomes upregulated on monocytes/macrophages leading to a lowering of antibody-dependent effector capacity(57–60) whilst in contrast enhancing the potential for greater costimulatory agonism.(34) Therefore, the proportions of various myeloid cells in the TME and their relative activating to inhibitory FcγR levels will influence the mechanisms by which agonist mAb stimulate an anti-tumor response.

One approach seeking to take advantage of this phenomenon and overcome the relatively low affinity of human IgG isotypes for FcγRIIB is to use Fc engineering to selectively enhance mAb engagement with FcγRIIB using a number of established mutations. (51,61) This has been shown to work in vitro for several TNFR including CD40(40), OX40(40) and CD27(43). Although proof of concept has been demonstrated in pre-clinical models, intratumoral administration is required to overcome elevated toxicity.(62,63) Therefore, alternative strategies have attempted to overcome this, for example by replacing the FcγR-mediated cross-linking with an alternative receptor, such as a tumor associated antigen (TAA)(64–66) or avoiding activation of cell types associated with toxicity (macrophages and monocytes) by targeting receptors on dendritic cells (67,68). Such approaches are exciting with some showing clinical promise.(69,70) but it should be noted that their development also involves additional complexity in design, production and biological insight to elicit effective targeting and receptor activation.

In contrast, FcγR-independent solutions, such as through hIgG2, may provide a tractable approach for some agonist antibodies. They can function independently of hFcγR and so for some molecules and targets might better support systemic delivery, simplifying the route of administration and providing mAb access to sites outside the TME, for example where a TAA-bispecific may be restricted (e.g. draining lymph nodes for T cell priming etc.).

Also, as described above, Tregs may also be an important determinant of efficacy of agonist mAbs targeting T cell costimulatory receptors, such as 4-1BB, OX40 and GITR. Several studies demonstrate that these molecules are preferentially overexpressed on Treg in a range of human tumors.(71,72) In pre-clinical models, mAb targeting costimulatory receptors and engaging activating FcγR can deplete intratumoral Tregs and elicit anti-tumor CD8 T cell responses.(73) This TME effect clearly complicates the targeting of these molecules as should the mAb induce agonism of Tregs rather than depletion this could lead to their activation and expansion, thus restricting the anti-tumor response. Subsequent pre-clinical studies showed that for optimal activity in mice, mIgG1 can directly activate costimulatory receptor expressing CD8s when sufficient inhibitory FcγRIIB is present on tumor myeloid cells, whereas mIgG2a/c are most effective when target expression is high on intratumoral Treg and activating FcγR dominate the TME (73–75). Unfortunately, varying levels of both activating and inhibitory FcγR are commonly present in tumors and so the outcome is more difficult to predict, especially in humans. Moreover, often the potential stimulatory impact of the agonist mAb on the intratumoral Treg is often overlooked, and it is unclear whether it negatively contributes to outcome. Given that CD8 agonism and Treg depletion have opposing FcγR engaging requirements and show competition for FcγR availability, blunting their efficacy, the potential to specifically engage one of these mechanisms without inducing undesirable effects with the other are challenging. One means to do this and combine both mechanisms simultaneously is to disconnect the FcγR engaging requirement from one or both of these approaches.(74) This was previously achieved by grafting the agonistic hIgG2B hinge into a high activating:inhibitory FcγR, depleting mAb isotype (i.e. mIgG2a). This permitted preferential depletion of 4-1BB-high intratumoral Treg and FcγR-independent agonism of 4-1BB-low expressing CD8 T cells. Although this approach was successful pre-clinically whether it would be robust in clinical applications is an open question.

In summary, the TME can alter the expression of both costimulatory targets and FcγR that can be required to engage them through agonism or depletion in a cell-type specific manner. Understanding these factors and identifying patients with suitable expression profiles(76) and related effector mechanisms will be critical to effectively harness these agonist approaches. Furthermore, a recent study from Amit and colleagues (77) demonstrated that activating FcγR-mediated effector cell signaling could also reprogram the TME and enhance anti-tumor immune responses. Therefore, whether the pragmatic approach to avoid engaging FcγR and for example, utilize TAA-mediated clustering, means that potential beneficial FcγR-mediated signaling effects are lost remains to be determined. Ongoing efforts to further our understanding of FcγR biology may serve to clarify these issues. For now, the clinical promise being observed with TAA-bispecifics serve to keep the future of these preclinically powerful agents very much alive.

## Clinical experience with agonists to date

To date, several agonist antibodies have been assessed clinically (Table 1). Here, we provide a summary of the clinical findings focussing on trials that include systemically administered mAb that target members of the TNFRSF as monotherapies; the latter to ensure the observed clinical effects are directly attributable to the agonists.

## CD40

CP870, 893 has a maximum tolerated dose (MTD) 10-40 fold lower than other anti-CD40 mAbs, ChiLob 7/4 and dacetuzumab (0.2 versus 3.3 versus 8 mg/kg), which are both hIgG1. The side effects observed with CD40 agonists were mostly mild-to-moderate but a few dose-limiting toxicities (DLTs) were noted, in particular, cytokine release syndrome, ocular toxicity and hepatotoxicity. These are hypothesized to be the result of on-target binding to CD40 on endothelium, conjunctiva and liver macrophages. Most events were transient and self-limiting. The anti-tumor efficacy with anti-CD40 agonists in these early-phase, dose-escalating monotherapy studies were modest, ranging from objective response rates (ORR) of 0-10% and stable response rates of 10-40%. Whether direct-targeting mechanisms or agonism were behind the observed anti-tumor efficacy are unclear. CD40 is expressed on B-cell lymphoid tumors and a proportion of solid tumors but the level of CD40 expression on tumor cells did not correlate with clinical responses. Tumor regression was also observed in metastatic melanoma lesions with CP870, 893 where CD40 is less likely to be expressed, altogether suggesting that agonism contributes to efficacy.(78) Transient peripheral blood B-cell depletion and upregulation of CD86 on residual B cells were observed in some participants, alongside elevation of inflammatory cytokines.

Whilst not reported within a monotherapy setting, the agonistic anti-CD40, sotigalimab, merits discussion as an example of ongoing clinical investigation. The PRINCE study randomized 99 participants with metastatic pancreatic adenocarcinoma to sotigalimab and chemotherapy (gemcitabine and nab-paclitaxel) versus nivolumab (anti-PD1) and chemotherapy vs sotigalimab, nivolumab and chemotherapy in first-line treatment.(79) Significant improvement in 1-year overall survival was observed with nivolumab and chemotherapy against a historical cohort (57.7% vs 35%) but not in the other two arms with the progression-free survival curves overlapping. Paired biopsies in two out of three participants showed increased tumor-infiltrating macrophages after sotigalimab/chemotherapy administration which was absent in other arms. Increased circulating numbers of Ki67+ non-naïve T-cells and IFNγ, CXCL9 and CXCL10 was observed across all arms, and interestingly, at an earlier time point for nivolumab-containing arms than sotigalimab (e.g. 2 weeks after treatment as opposed to 4-16 weeks).

## 4-1BB

Utomilumab is a hIgG2 mAb targeting 4-1BB. Unlike CP870, 893, it is a weak agonist, particularly in comparison to the hIgG4 urelumab (whose MTD is 100-fold lower). The clinical response rate for urelumab above 1 mg/kg has not been reported but it induced severe hepatotoxicity and resulted in two deaths at this level. Urelumab and utomilumab (administered <1 mg/kg and ≤10 mg/kg, respectively) had modest disease control rate (i.e. combined complete, partial and stable response rate). The distinct potency between the two mAbs is now understood to be due to differences in epitope and isotype. (36) Whilst 4-1BB is not highly expressed in the liver, FcγRIIb, which is preferentially bound by hIgG4 compared to hIgG2, is highly expressed by Kupffer cells. The combined effect of urelumab’s non-ligand blocking, membrane-distal binding epitope to 4-1BB on CD8+ T cells in the liver and hyper-crosslinking of 4-1BB by FcγRIIb on Kupffer cells has been shown by several groups to induce the observed hepatotoxicity.(80,81)

## OX40

MEDI6469, the first OX40 agonist to enter clinical development, was deployed with as a murine IgG1. No objective responses or DLTs were observed in the phase I study in patients with advanced solid tumors, but the ability of MEDI16469 to elicit in vivo agonism was demonstrated by increased tumor-specific T- and B-cell responses in two patients with melanoma, and improved tetanus vaccine antibodies in a larger group of participants. Ivuxolimab, is an anti-OX40 hIgG2 agonist which displayed modest clinical efficacy in advanced stage cancers in the phase I study. Its dose was escalated to 10 mg/kg and no DLTs were observed. OX40 expression in the peripheral blood was heterogeneous and as expected, predominantly on CD4+ central and effector memory T cells. Some evidence of in vivo agonism was observed - paired tumor biopsies obtained at baseline and 6 weeks after treatment in participants dosed ≥ 1.5 mg/kg showed enrichment of inflammatory and immune activation gene signatures. The association between these signatures and clinical response is unclear. Only 1/29 participants experienced a partial response. Two other participants that experienced a partial response at lower dosing were not biopsied. The remaining published OX40 agonists are hIgG1 mAbs. This class of reagents have been largely well-tolerated and the MTD undefined but again, with modest clinical efficacy.

## GITR

The clinically explored mAbs against GITR are all hIgG1 molecules. TRX518 is further aglycosylated for enhanced affinity for FcγRIII and augmented depleting ability. Similar to OX40 hIgG1 mAbs, these drugs have been well-tolerated with the MTD undefined. Low objective response rates are observed, ranging from 0-3%, but with higher stable disease rates (18-70%). TRX518-treated participants who achieved stable disease had reduced intratumoral Tregs compared to baseline biopsies, in contrast to participants with progressive disease, where an increase in intratumoral Tregs were observed. (82)

## CD27

Varlilumab, a hIgG1 CD27 agonist was also selected with the aim of simultaneously mediating Treg depletion and agonism. Reduction in the peripheral CD4+ T-cell population including Tregs were observed in the phase I studies alongside a transient increase in peripheral inflammatory cytokines. A few participants with melanoma also showed increased CD8+ T-cell reactivity to melanoma antigens. Despite this, ORR was <5% and stable disease rate <20%. One DLT was observed (grade 3 hyponatremia) but otherwise varlilumab was well-tolerated. A further hIgG1 agonist (MK-5890) is currently undergoing clinical testing in advanced solid tumors but the mature data is yet to be published.(83)

## DR5

DR5 is expressed on a wide range of haematopoietic and solid tumors. Therefore, distinct to the molecules above, DR5 agonists rely on the induction of direct tumor cell death. The ability of these agonists to induce objective responses has been low (<10%) although the tetravalent hIgG1 molecule, INBRX-109, induced stable disease in 80% of chondrosarcoma cases. In vivo evidence of DR5 agonism is lacking and often unreported. When conatumumab (an unmodified hIgG1 anti-DR5) was examined, intratumoral caspase-3 activation was observed in 2/7 cases, but not associated with clinical response(84). Hepatotoxicity was observed with both INBRX-109 and drozitumab (hIgG1). Two participants died of liver failure with INBRX-109 but this may have been accounted for, or aggravated by, other factors. A further 4/31 participants experienced grade 2/3 elevation of hepatic enzymes with drozitumab. The observed hepatotoxicity is likely to be due to on-target binding of the agonist to DR5-expressing hepatocytes.

These early phase trials described above primarily aimed to assess the safety rather than clinical efficacy of the agonist antibodies. Regardless, the efficacy signal in in these monotherapy trials has been underwhelming with limitations in efficacy/activity or issues with toxicity. As such, attention has shifted towards combination with other agents or development of newer antibody formats. These modified antibodies have been readily adopted by the field and a second wave of ‘synthetic’ TNFRSF agonists have followed the classic bivalent mAb based approaches. These ‘second generation’ synthetic molecules are often non-FcγR dependent agonists which fall into two main classes; single valency TNFRSF bi/trispecifics that co-engage TAA or immune checkpoint molecules to deliver cross-linking and consequently higher-order clustering, and multivalent TNFRSF agents constituted of antibody-based molecules or ligand:antibody hybrids with intrinsic clustering potential. Interestingly, both these classes of synthetic agonist have, in general, shown less evidence of the cytokine release syndrome and increased liver enzymes associated with their bivalent antibody agonist predecessors. This may indicate that these non-FcγR dependent approaches have decoupled dose limiting toxicity from effective agonism as suggested in pre-clinical studies.(85) However, many of these molecules are still in the early stages of clinical investigation and are yet to report fully and so we must await definitive findings in this regard. Further detailed discussion of these synthetic agonist strategies is outside the scope of this review but they are evolving rapidly and have been reviewed recently conceptually(86) and with 4-1BB targeting as an exemplar.(69)

## Conclusion

The earlier success of tumor-targeting antibodies like rituximab and the PD-1/PD-L1 antibodies drove academia and pharma to explore new antibody targets, including agonist antibodies. This led to a flurry of clinical trials involving these agonists, none of which have demonstrated significant clinical efficacy. However, the antibody formats chosen were perhaps not optimal, with understanding of epitope and isotype lacking at the time, coupled to under-appreciation of the impact of the TME. In addition, their kinetics of response compared to checkpoint blockade as indicated in the PRINCE study discussed above is an interesting aspect. That agonists might take longer to induce tumour reduction may require a reconsideration of conventional trial designs, even when pseudo progression is taken into account.

Despite the agonists’ failure to induce significant clinical responses, much has been learned from the early phase monotherapy trials and following pre-clinical work. Unlike immune checkpoint inhibitors, where diverse immune effects are observed, these have been less frequent with agonist antibodies. Instead, hepatotoxicity secondary to on-target toxicity has been the commonest safety concern. Hepatotoxicity is closely linked to agonistic potency, thus measures to broaden the therapeutic window will be important, including through bispecific and other approaches. One means of achieving this by localising the antibody in the tumor, such as exemplified by GEN1046, an antibody targeting 4-1BB and PD-L1, wherein the MTD was not reached at a dose >10 mg/kg and the safety profile was manageable.(87)

Another potentially important consideration for future clinical development is patient and/or cancer selection. Most of the early phase trials have recruited ‘all comers’ with advanced, multiply treated disease, and have not discriminated between cancer type on the basis that host immunity is being targeted instead of the tumor cells. Nevertheless, sufficient expression of the target in the tumor is likely to be important to enable the threshold for agonism to be reached; this is likely to differ between patients and tumor types and may necessitate targeting of multiple receptors to achieve the desired level of immune stimulation. Similarly, as described above, the nature of the target, FcγR expression pattern, TME composition and desired mechanism of action will all play their part. As we learn more about these aspects our ability to successfully leverage agonist antibodies in the clinic will surely follow.

## Figures and Tables

**Table 1:** Safety, efficacy and evidence of in vivo agonism in phase I-II agonist monotherapy trials in cancer.

Target	mAb	Company	Isotype	Phase (Trial ID)	Disease population, N	Complete/ partial responses	Stable response rate (n, %)	Safety	MTD* or tolerable dose (mg/kg)	Evidence of in vivo agonism	Reference
CD40	**SEA-CD40**	Seattle Genetics	Non-fucosylated hIgG1	Phase I (NCT02376699)	Advanced cancers, 67	5 2(3.0%)	9 (13.4%)	No DLT	0.045-0.060	B-cell depletion, transient T-cell and NK activation and depletion, transient cytokine activation (IP-10, MIG, MIP1β) in peripheral blood.	(88)
	**CP870,893**	Pfizer	hIgG2	Phase I	Advanced solid malignancies, 29	4 (13.8%)	7 (24%)	DLT (thromboembolism, headache)	0.2*	B-cell depletion and activation (CD86) in peripheral blood	(78,89)
				Phase I (NCT02157831)	Advanced solid malignancies, 27	0 (0%)	7 (26%)	DLT (grade 3 cytokine release and urticaria)			(78,89)
	**Mitazalimab**	Alligator Bio-science	hIgG1	Phase I (NCT02829099)	Advanced solid malignancies, 95	1 (1.1%)	35 (36.8%)	2 DLT (grade 3 headache and hepatotoxicity)	0.9	Transient increase in MCP-1, IP-10 and MIP-1β and transient reduction in B, NK and T cells in peripheral blood	(90)
	**ChiLob7/4**	University of Southampton	hIgG1	Phase I (NCT01561911)	Advanced cancers, 28	0 (0%)	15 (53.6%)	DLT (liver transaminase elevation)	2.1-3.3*	Transient increase in MIP1β in peripheral blood	(91)
	**Dacetuzumab**	Seattle Genetics	hIgG1	Phase I (NCT00103779)	B-NHL, 50	6 (12%)	13 (26%)	2 DLT (grade 3 conjunctivitis and transient vision loss, grade 3 ALT elevation)	4-8	Transient increase in IL-1β, IL-6, IL-10, TNFα in peripheral blood	(92)
				Phase I (NCT00283101)	CLL, 12	0 (0%)	5 (41.7%)	1 DLT (grade 4 thrombocytopenia)			(93)
				Phase II (NCT00435916)	DLBCL, 46	4 (9%)	13 (37%)	Grade 3/4 events observed (ocular toxicity (2), transaminitis (1), cytokine release syndrome (1)		Not examined	(94)
				Phase I (NCT00079716)	Multiple myeloma, 44	0 (0%)	9 (20%)	DLTs observed (cytokine release syndrome, ocular toxicity, transaminitis.		Not examined	(95)
**CD27**	**Varlilumab**	Celldex Therapeutics	hIgG1	Phase I (NCT01460134)	Advanced solid tumors, 56; Lymphomas, 34	Solid tumors: 1 (1.8%); Lymphomas: 1 (2.9%)	Solid tumors: 8 (14.3%); Lymphomas: 3 (8.8%)	1 DLT (grade 3 transient hyponatremia)	10	Transient increase in proinflammatory cytokines, Treg depletion and augmentation of T-cell reactivity (in a subset of participants) in peripheral blood	(96,97)
**4-1BB**	**Urelumab**	Bristol-Myers Squibb	hIgG4 (S228P hinge mutation)	Phase I (NCT00309023, NCT00612664, NCT01471210)	B-NHL (NCT01471210), 60; Advanced solid tumors, 347	6 (10%) (B-NHL)	17 (28%) (B-NHL)	Hepatotoxicity; 2 deaths (n=347)	0.1*	IFN-induced cytokines and IFN-response genes in peripheral blood	(98,99)
	**Utomilumab**	Pfizer	hIgG2	Phase I (NCT01307267)	Advanced solid tumors, 55	4 (7.3%)	13 (24.5%)	No DLT	10	No consistent evidence in peripheral blood.	(100)
**GITR**	**GWN323**	Novartis	hIgG1	Phase I/Ib (NCT02740270)	Advanced solid tumors, 39	0 (0%)	7 (17.9%)	No DLT	~21 (1500 mg flat dose)#	No evidence in peripheral blood or tumor	(101)
	**MK-4166**	MSD	hIgG1	Phase I (NCT02132754)	Advanced solid tumors, 48	0 (0%)	11 (22.9%)	DLT in 1 participant (grade 3 bladder perforation)	~12.9 (900 mg flat dose)	Not examined	(102)
	**TRX518**	Leap Therapeutics	Aglycosylated hIgG1	Phase Ib (NCT02628574)	Advanced solid tumors, 43 (31 evaluable)	1 (3.2%)	22 (71.0%)	No DLT	1 (4 mg/kg loading dose)	Treg reduction in periphery;Treg reduction and CD8 and Granzyme B increase in clinical responders.	(82)
	**AMG 228**	Amgen	IgG1	Phase I (NCT02437916)	Advanced solid tumors, 30 (27 evaluable)	1 (0%)	7 (23%)	No DLT	~ 17.1 (1200 mg flat dose)	No evidence in peripheral blood	(103)
	**BMS-986156**	Bristol-Myers Squibb	hIgG1	Phase I (NCT02598960)	Advanced solid tumors, 34	0 (0%)	11 (32.3%)	No DLT	~11.4 (800 mg flat dose)	Trend to increased proinflam cytokines in peripheral blood; no evidence in tumor.	(104, 105)
**OX40**	**MEDI6469**	Med-immune	murine IgG1	Phase I (NCT01644968)	Advanced solid tumors, 30	1 (0%)	12 (40%)	No DLT	2	Increased peripheral T and B cell vaccine reactivity and OX40 upregulation on Treg in tumor	(106)
				Phase Ib (NCT02274155)	HNSCC, 17	n/a Neoadjuvan t prior to surgical resection	n/a	No DLT		Increased CD4+ and CD8+ T cells in peripheral blood and tumor	(107)
	**PF04518600 (Ivuxolimab)**	Pfizer	hIgG2	Phase I (NCT02315066)	Advanced cancers, 52	3 (5.6%)	26 (56%)	No DLT	10	Increased profilferation of CD4 CM and EM cells in some participants in PB; Increased immune cell infiltration and immune activation/inflammation gene sets in tumor.	(108)
	**BMS986178**	Bristol-Myers Squibb	hIgG1	Phase I/IIa (NCT02737475)	Advanced cancers, 20	0 (0%)	7 (35%)	No DLT	~4.6 (320 mg flat dose)	Not examined	(109)
	**MOXR0916 (Vonlerolizu-mab)**	Genentech	hIgG1	Phase I (NCT02219724)	Advanced solid tumors, 174	2 (1.1%)	113 (66%)	No DLT	~17 (1200 mg flat dose)	Immune activation in tumor	(110)
	**MEDI0562 (Tavolimab)**	AstraZeneca	hIgG1	Phase I (NCT02318394)	Advanced solid tumors, 55 (50 evaluable)	2 (4.0%)	22 (44%)	1 DLT (diarrhoea)	10	Increased peripheral Ki67+ CD4 and C8 memory T cells; decreased intratumoral OX40+ Treg	(111)
	**INCAGN-01949**	Incyte Corporation	hIgG1	Phase I (NCT02923349)	Advanced solid tumors, 87	1 (1.1%)	23 (26.4%)	1 DLT (grade 3 colitis)	~20 (1400 mg flat dose)	No evidence in peripheral blood or tumor	(112)
**DR5**	**PRO95780 (Drozitumab)**	Genen-tech	hIgG1	Phase I	Advanced cancers, 50 (41 evaluable)	0 (0%)	20 (49%)	2 DLTs (grade 4 pulmonary embolism and grade 3 serum ALT elevation)	20	Not examined	(113)
	**INBRX-109**	Inhibrx, Inc.	Tetravalent hIgG1	Phase I (NCT03715933)	Metastatic chondrosarcoma, 31	2 (6.5%)	25 (80.6%)	No DLT; but 2 deaths due to hepatic failure	3	Not examined	(114)
	**CS-1008 (Tigatuzumab)**	Daiichi Sankyo, Inc.	hIgG1	Phase I (NCT01220999)	Metastatic colorectal carcinoma, 19	1 (5.3%)	8 (42.1%)	No DLT	2	No evidence in peripheral blood or tumor	(115)
	**DS-8273a**	Daiichi Sankyo, Inc.	hIgG1	Phase I (NCT02076451)	Advanced cancers, 32	0 (0%)	10 (31.3%)	No DLT	24	Decreased in peripheral myeloid derived suppressor cells	(116)
	**Conatumumab**	Amgen	hIgG1	Phase I	Advanced solid tumors, 37	1 (2.7%)	14 (37.8%)	No DLT	20	No evidence in tumor	(84)

# The flat dose is estimated based on a 70 kg participant weight

Complete/partial response rate(%)
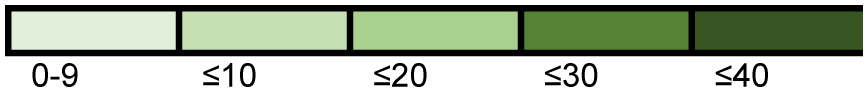

Stable response rate (%)
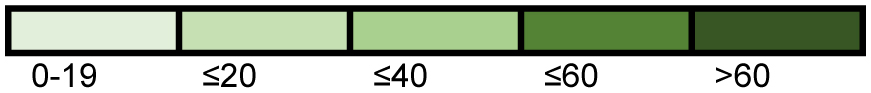

MTD/tolerable dose(mg/kg)
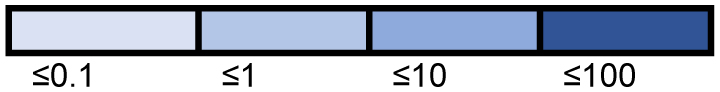

DLTs